# *Journal of Experimental Botany* 70th anniversary: plant metabolism in a changing world

**DOI:** 10.1093/jxb/erab352

**Published:** 2021-09-02

**Authors:** Robert D Hancock, Nicholas Smirnoff, John E Lunn

**Affiliations:** 1 Cell and Molecular Sciences, The James Hutton Institute, Dundee DD2 5DA, UK; 2 Biosciences, College of Life and Environmental Sciences, University of Exeter, Exeter EX4 4QD, UK; 3 Max Planck Institute of Molecular Plant Physiology, D-14476 Potsdam-Golm, Germany

**Keywords:** Calvin–Benson cycle, C_3_ photosynthesis, C_4_ photosynthesis, modelling, Rubisco, secondary metabolism, sucrose-non-fermenting-1-related protein kinase 1, thioredoxin

**Environmental change is a fact of life for plants and occurs over different time frames, ranging from second-by-second fluctuations in light levels to changes in atmospheric composition and global temperatures occurring over millions of years. Being able to adapt to environmental change is essential not only for the survival of individual plants, but also for the long-term success of plant species. In this special issue, which celebrates the 70th anniversary of the founding of the Journal of Experimental Botany in 1950, we explore how plants adapt their metabolism to environmental challenges and how this knowledge might help us to improve crop resilience to global climate change.**

Photosynthesis is the fundamental source of carbon and energy for plants, and indeed almost all living organisms. This process is particularly exposed to environmental fluctuations, being directly dependent on light, CO_2_ concentration, water availability, and temperature. Other environmental factors, such as nutrient supply, also have an indirect impact on photosynthesis by affecting the plant’s ability to build the proteins, pigments, and cofactors that form its photosynthetic apparatus. Most plants perform C_3_ photosynthesis via the Calvin–Benson cycle, with initial fixation of CO_2_ by the enzyme Rubisco into a three-carbon compound, 3-phosphoglycerate. The pathway is thought to have appeared in anoxygenic green bacteria and purple bacteria >2 billion years ago, when the atmosphere was reducing and rich in CO_2_. The evolution of oxygenic photosynthesis in proteobacteria and cyanobacteria, ~2 billion years ago, led to the appearance of molecular oxygen in the atmosphere, sometimes referred to as the ‘great oxidation event’ or ‘oxygen catastrophe’. This fundamentally changed conditions for life on earth and exposed a basic flaw in the enzymatic activity of Rubisco, namely its side reaction with oxygen. This not only competes with the carboxylation reaction but also generates a toxic product, 2-phosphoglycolate, which needs to be detoxified and recycled via the pathway of photorespiration. Various carbon-concentrating mechanisms evolved in some groups of photosynthetic organisms, including cyanobacteria (carboxysomes) and eukaryotic algae (pyrenoids). These mechanisms promote the capture of carbon in aquatic and other CO_2_-poor environments, and minimize the competing oxygenation reaction of Rubisco, thereby suppressing photorespiration.

In their review, Stitt *et al.* (2021) explore unexpected diversity in the operation of the Calvin–Benson cycle in different C_3_ species, reflecting divergent paths in different plant lineages as they evolved and adapted to changes in atmospheric CO_2_ concentrations and other environmental parameters. Flux through the Calvin–Benson cycle is regulated at many levels, with the activity of several enzymes in the pathway being modulated by reversible conversion between their dithiol and disulfide forms, mediated by thioredoxins. This redox modulation of chloroplastic enzymes is particularly important because it helps to coordinate flux of carbon with the capture of light energy, which provides the ATP and reducing power (NADPH) needed for CO_2_ fixation. da Fonseca Pereira *et al.* (2021) describe how thioredoxin-mediated redox regulation extends beyond the chloroplast to other enzymes and cellular compartments involved in photorespiration, and is also important in regulation of respiratory pathways that provide energy and building blocks for growth. Using modelling approaches, Iqbal *et al.* (2021) explore how Rubisco limits the efficiency of C_3_ photosynthesis in crop canopies and discuss prospects for improving the catalytic efficiency of Rubisco to enhance C_3_ crop yields.

About 100–150 million years ago, atmospheric CO_2_ levels declined in parallel with changes in global temperatures and rainfall that constrained C_3_ photosynthesis even further. It is thought that this selective pressure favoured the independent evolution of another carbon-concentrating mechanism—C_4_ photosynthesis—in >60 plant lineages (Sage, 2004). In this pathway, CO_2_ is initially fixed via phosph*enol*pyruvate carboxylase into a four-carbon compound, oxaloacetate, which is then converted to malate or aspartate in specialized mesophyll cells. These C_4_ acids diffuse into the bundle sheath cells, where they are decarboxylated to release CO_2_ which is refixed by Rubisco via the Calvin–Benson cycle, while the remaining C_3_ moiety returns to the mesophyll cells to be recycled as the acceptor for initial CO_2_ fixation. The C_4_ cycle acts as a biochemical CO_2_ pump that raises the concentration of CO_2_ in the bundle sheath cells, thereby suppressing the oxygenase activity of Rubisco and the need for photorespiration. This pathway also confers greater water and nitrogen use efficiencies on C_4_ plants. von Caemmerer (2021) introduces an updated and parameterized model for C_4_ photosynthesis that includes both biochemical and biophysical aspects of the pathway. This model will enable researchers to better understand how the pathway operates and how it responds to changing environmental conditions, such as temperature. This knowledge will be invaluable for those seeking to engineer the pathway to optimize its operation in C_4_ crop species, such as maize (*Zea mays*), sugarcane (*Saccharum officinarum*), and sorghum (*Sorghum bicolor*), and to introduce it into C_3_ crop species, such as rice (*Oryza sativa*), to improve their photosynthetic efficiency and productivity.

Although C_4_ species generally outperform C_3_ species, especially in warmer regions, there may still be scope for improving the efficiency of photosynthesis in C_4_ crop plants. Using modelling approaches, Sales *et al.* (2021) identify potential inefficiencies in NADP-malic enzyme-type C_4_ photosynthesis under non-stressed conditions and additional constraints on the pathway when plants are exposed to drought, chilling, or high temperature stress, and propose various strategies to overcome these limitations. Introducing C_4_ photosynthesis into C_3_ crop plants, such as rice (*Oryza sativa*), has the potential to increase photosynthetic rates and crop yields by up to 50% (https://c4rice.com/). However, this ambitious goal faces many technical challenges as it requires not only the introduction of C_4_ biochemistry, but also modifications of leaf anatomy, including changes in leaf venation and development of specialized photosynthetic mesophyll and bundle sheath cells. The enzymes, transporters, and regulatory factors needed to operate a C_4_ photosynthetic cycle also need to be precisely targeted to the right cell type if the pathway is to operate efficiently. Furbank and Kelly (2021) describe how the division of labour between mesophyll and bundle sheath cells extends to pathways downstream of carbon fixation, namely the synthesis, storage, and export of carbohydrates. Starch synthesis and storage occur almost exclusively in the bundle sheath cells, while sucrose is usually made predominantly in mesophyll cells, but must pass through the bundle sheath cells to be loaded into the phloem. In their review, Furbank and Kelly (2021) explore the reasons for this division of labour, and whether this is a consequence of the compartmentation of C_4_ photosynthesis or an essential requirement for C_4_ photosynthesis, with implications for the engineering of C_4_ photosynthesis in C_3_ plants.

Photosynthetic carbon fixation in the leaves provides the energy and carbon needed not only for growth, but also for defence against pathogens and adaptations to abiotic stress. Therefore, plant growth is susceptible to environmental factors that affect the supply of photoassimilates, and to biotic and abiotic stresses that divert resources to defence. Plants synthesize an extraordinary range of secondary metabolites, collectively >1 million by some estimates, many of which help to defend the plant against microbial pathogens, herbivores, excess light, UV radiation, and other sources of oxidative stress. Panda *et al.* (2021) review recent progress in understanding how plants manage the delicate balancing act between growth and defence by tightly regulating the production of defence compounds, and the strategies employed by plants to protect themselves from the cytotoxic compounds that they synthesize to defend themselves. Jamsheer *et al.* (2021) explore the other side of the coin, in their review of a key regulator of plant growth—SNF1-related protein kinase 1 (SnRK1). This trimeric protein kinase belongs to a conserved family of protein kinases found in all eukaryotes, and serves as a central hub for sensing the metabolic status of the plant and regulating its growth and development accordingly.

Plants have clearly evolved over deep time (for example, by developing carbon-concentrating mechanisms as discussed above) to deal with climate change and changes in atmospheric CO_2_. However, the rapid pace of global climate change linked to rising atmospheric CO_2_ levels poses an unprecedented challenge to agriculture as well as driving rapid changes in species distribution in natural ecosystems. If current trends continue, predicted rises in temperature and more erratic rainfall will have a severely negative impact on agricultural productivity, at a time when we need to increase food production to support a growing human population. All aspects of plant life are influenced by their environment, but it is their metabolism that is most directly affected by short- and long-term environmental changes, which will then have an impact on their growth and development. Photosynthesis is key to biomass gain, but fine-tuning and integrating primary and secondary metabolism in response to changing environmental pressures may also be key to more sustainable agricultural practices. The advances in our understanding of the metabolic processes that underlie plant productivity and defence described in this special issue will contribute to identifying traits that will improve the climate resilience of crops and will enable improved prediction of upcoming changes in natural ecosystems.

## References

[CIT0001] da Fonseca-Pereira P, Souza PVL, Fernie AR, Timm SL, Daloso DM, Araújo WL. 2021. Thioredoxin-mediated regulation of (photo)respiration and central metabolism. Journal of Experimental Botany 72, 5987–6002. 33649770 10.1093/jxb/erab098

[CIT0002] Furbank RT, Kelly S. 2021. Finding the C_4_ sweet spot: cellular compartmentation of carbohydrate metabolism in C_4_ photosynthesis. Journal of Experimental Botany 72, 6018–6026. 34142128 10.1093/jxb/erab290 PMC8411606

[CIT0003] Iqbal WA, Miller IG, Moore RL, Hope IJ, Cowan-Turner D, Kapralov MV. 2021. Rubisco substitutions predicted to enhance crop performance through carbon uptake modelling. Journal of Experimental Botany 72, 6066–6076. 34115846 10.1093/jxb/erab278 PMC8411856

[CIT0004] Jamsheer KM, Kumar M, Srivastava V. 2021. SNF1-related protein kinase 1: the many-faced signaling hub regulating developmental plasticity in plants. Journal of Experimental Botany 72, 6042–6065. 33693699 10.1093/jxb/erab079

[CIT0005] Panda S, Kazachkova Y, Aharoni A. 2021. Catch-22 in specialized metabolism: balancing defense and growth. Journal of Experimental Botany 72, 6027–6041. 34293097 10.1093/jxb/erab348

[CIT0006] Sage RF. 2004. The evolution of C_4_ photosynthesis. New Phytologist 161, 341–370. 10.1111/j.1469-8137.2004.00974.x 33873498

[CIT0007] Sales CRG, Wang Y, Evers JB, Kromdijck J. 2021. Improving C_4_ photosynthesis to increase productivity under optimal and suboptimal conditions. Journal of Experimental Botany 72, 5942–5960. 34268575 10.1093/jxb/erab327 PMC8411859

[CIT0008] Stitt M, Borghi GL, Arrivault S. 2021. Targeted metabolite profiling as a top-down approach to uncover interspecies diversity and identify key conserved operational features in the Calvin–Benson cycle. Journal of Experimental Botany 72, 5961–5986. 34473300 10.1093/jxb/erab291 PMC8411860

[CIT0009] von Caemmerer S. 2021. Updating the steady-state model of C_4_ photosynthesis. Journal of Experimental Botany 72, 6003–6017. 34173821 10.1093/jxb/erab266 PMC8411607

